# Benzodiazepines and Z-Drug Use among HIV-Infected Patients in Taiwan: A 13-Year Nationwide Cohort Study

**DOI:** 10.1155/2015/465726

**Published:** 2015-01-13

**Authors:** Han-Ting Wei, Mu-Hong Chen, Wing-Wai Wong, Yuan-Hwa Chou, Ying-Jay Liou, Tung-Ping Su, Tzeng-Ji Chen, Ya-Mei Bai

**Affiliations:** ^1^Department of Psychiatry, Taipei Veterans General Hospital, Yuanshan Branch, Yilan County, Taiwan; ^2^Department of Psychiatry, Taipei Veterans General Hospital, No. 201, Sec. 2, Shipai Road, Beitou District, Taipei City 11217, Taiwan; ^3^Division of Infectious Disease, Department of Medicine, Taipei Veterans General Hospital, Taipei City, Taiwan; ^4^Department of Psychiatry, College of Medicine, National Yang-Ming University, Taipei City, Taiwan; ^5^Department of Family Medicine, Taipei Veterans General Hospital, Taipei City, Taiwan

## Abstract

*Introduction*. Benzodiazepines (BZDs) and zolpidem, zopiclone, and zaleplon (Z-drugs) are commonly prescribed to HIV-infected patients. We hypothesized that frequent BZD and Z-drug use among these patients may be associated with psychiatric illnesses, particularly in long-term users. *Methods*. We included 1,081 patients with HIV between 1998 and 2011 from the Taiwan National Health Insurance Research Database and matched them according to age, sex, and comorbidity with uninfected controls to investigate the psychiatric diagnoses and prescriptions of BZDs and Z-drugs. Cumulative defined daily dose (cDDD) was assessed as the indicator of the duration of medication exposure. Patients exhibiting a cDDD exceeding 180 were defined as long-term users. *Results*. The patients with HIV had an increased risk of any use (odds ratio (OR): 8.70, 95% confidence interval (CI): 6.82–10.97) and long-term use (OR: 5.06, 95% CI: 3.63–7.04) of BZD and Z-drugs compared with those without HIV during the follow-up after demographic data and psychiatric comorbidities were adjusted. *Conclusion*. A large proportion of the HIV-infected patients received prescriptions of BZDs and Z-drugs. Mood disorders, insomnia, anxiety disorders, HIV infection, and substance use disorder were substantial predictors among the BZD and Z-drug users. These findings suggest that providing psychiatric services for HIV-infected patients is vital.

## 1. Introduction

Insomnia and anxiety are common complaints among patients infected with HIV [1–3] who live with the stress of a highly stigmatized and potentially life-threatening physical illness. These patients experience a considerable psychological burden that results in a high prevalence of insomnia, anxiety, and depression [4, 5]. Rubinstein and Selwyn [6] reported that 73% of 115 HIV-infected outpatients experienced insomnia. Hudson et al. [7, 8] determined that 77% of 118 HIV-infected women experienced fear and worry, and insomnia and anxiety exerted the most noticeable influence on their daily lives. Therefore, hypnotic and anxiolytic medications such as benzodiazepines (BZDs) and zolpidem, zopiclone, and zaleplon (Z-drugs) are commonly prescribed to HIV-infected patients.

A large study evaluating the psychotropic medications of HIV-infected patients found that, among 1,489 patients, 16.7% received anxiolytics and 20.9% received antidepressants [9]. However, hypnotic and anxiolytic medications can be misused or be physically and psychologically addictive, increasing the risks of physical harm, dependence, and social harm [10]. Darke et al. [11] reported that BZD users exhibited high-HIV-risk behaviors, such as sharing injection equipment with numerous people and injecting themselves with multiple drugs. Furthermore, a new class of hypnotic Z-drugs reportedly has a considerably high prevalence of misuse, abuse, and dependence among the general population; however, few studies have reported the use pattern in an HIV-infected population [12].

In this study, we proposed that the high prevalence of BZD and Z-drug use may be associated with psychiatric illness, particularly in long-term users. Therefore, by using the Taiwan National Health Insurance Research Database (NHIRD) [13], we analyzed the use pattern of BZDs and Z-drugs among HIV-infected patients in Taiwan and associated factors.

## 2. Materials and Methods

### 2.1. Data Source

This study was based on data from the Taiwan NHIRD that was released by the National Health Research Institute (NHRI) [13]. The Taiwan National Health Insurance (NHI) program was implemented in 1995 and has insured 96.9% of the 23 million residents of Taiwan since 2001. The NHIRD contains information of insured residents, including age, sex, prescription drugs, prescription date, and diagnosis according to the International Classification of Diseases, Ninth Revision, Clinical Modification (ICD-9-CM). The NHRI has provided a database of the medical claims from 1 million random enrollees, approximately 4.3% of the population, for use in health service studies.

### 2.2. Participants

In this study, the HIV-infected patients in the database of 1 million random enrollees were identified using the ICD-9-CM codes 042, 043, 044 (HIV), and V08 (asymptomatic HIV infection status) for the period from January 1, 1998, to December 31, 2011. An age-, sex-, and comorbidity-matched (1 : 1) control cohortwas randomly identified from among 1 million enrollees after eliminating the study patients and those who were diagnosed with HIV at any time. The comorbidities of psychiatric disorders were defined as mood disorders (major depressive disorder, bipolar disorder, dysthymic disorder, and depressive disorder, not otherwise specified, ICD-9-CM codes: 296, 300.4, and 311), anxiety disorders (ICD-9-CM codes: 300, except 300.4), alcohol use disorders (ICD-9-CM codes: 303 and 305.00–305.03), substance use disorders (ICD-9-CM codes: 304 and 305; alcohol and smoking were excluded), and insomnia (ICD-9-CM codes: 307.4 and 780.5).

### 2.3. Definition of Drug Class

Three Z-drugs (zolpidem, zopiclone, and zaleplon) and common BZDs, such as midazolam, brotizolam, estazolam, flunitrazepam, nitrazepam, flurazepam, alprazolam, lorazepam, triazolam, diazepam, medazepam, bromazepam, clonazepam, fludiazepam, chlordiazepoxide, nordazepam, oxazolam, clorazepate, and nimetazepam, which are all permitted by the Taiwan Food and Drug Administration for treating insomnia and anxiety, were included in this study. We identified patients who filled prescriptions for BZDs and Z-drugs during the entire follow-up duration between the date of HIV-infection or enrollment and December 31, 2011. The defined daily dose (DDD) recommended by the World Health Organization is the assumed average maintenance dose per day for a drug used for its primary indication in adults and is a unit used for measuring a prescribed drug amount. We calculated the sum of the dispensed DDD of any BZD and Z-drug during the study period as an indicator of exposure duration. We defined the patients exhibiting a cumulative DDD (cDDD) exceeding 180 as long-term BZD and Z-drug users [14]. Furthermore, we assessed the different specialties of physicians (psychiatrists versus nonpsychiatrists) in relation to BZD and Z-drug prescriptions.

### 2.4. Statistical Analysis

In the between-group comparisons, an independent *t*-test was used for continuous variables and Pearson's *χ* ^2^ test was conducted for nominal variables, where appropriate. The logistic regression model was used to investigate the odds ratio (OR) and 95% confidence interval (CI) of any use or long-term use of BZDs and Z-drugs among the patients with HIV and the controls after demographic data and psychiatric comorbidities were adjusted. In addition, we identified the possible association of age at HIV infection, duration of HIV infection, and psychiatric comorbidities with any use or long-term use of Z-drugs and BZDs among the HIV-infected patients. A *P* value of less than 0.05 was considered statistically significant. All data processing and statistical analyses were performed using the Statistical Package for Social Sciences (SPSS) Version 17 (SPSS Inc.).

## 3. Results

Overall, 1,081 HIV-infected patients and 1,081 uninfected controls matched for age, sex, and comorbidity for the period from 1998 to 2011 were included. The subjects exhibited a mean age of 33.41 ± 13.81 years, and men were predominant (72.8%; Table 1). The HIV patients had a higher prevalence of any use (76.4% versus 38.6%, *P* < 0.001) and long-term use of BZDs and Z-drugs indicated by a cDDD exceeding 180 (30.5% versus 14.8%, *P* < 0.001) compared with the controls (Table 1). The HIV patients had a higher prevalence of any anxiety disorder (13.4% versus 11.2%, *P* = 0.033) and a lower income (*P* = 0.010) compared with those without HIV (Table 1). Among all of the BZD and Z-drug prescriptions between the HIV-infected patients and the controls, 31.1% and 34.3% were prescribed by psychiatrists and 68.9% and 65.7% by nonpsychiatrists, respectively.

In the logistic regression model, the adjustment of demographic data and psychiatric comorbidities showed that the HIV patients had an elevated risk of having any use (OR: 8.70, 95% CI: 6.82–10.97) or long-term use (OR: 5.06, 95% CI: 3.63–7.04) of BZDs and Z-drugs compared with the controls (Table 2). Insomnia (OR: 20.09, 95% CI: 10.49–38.47; OR: 10.69, 95% CI: 7.46–15.31), any mood disorder (OR: 9.86, 95% CI: 5.37–18.12; OR: 15.69, 95% CI: 10.18–24.17), and any anxiety disorder (OR: 7.24, 95% CI: 0.57–14.67; OR: 6.53, 95% CI: 4.34–9.82) were associated with an increased likelihood of having any use or long-term use of BZDs and Z-drugs (Table 2).

## 4. Discussion

The results of this study revealed that a large proportion of HIV-infected patients received BZD and Z-drug prescriptions. Among the HIV-infected patients, 76.4% were prescribed BZDs and Z-drugs at least once, an observation that supported the finding reported by Ochitill et al. that psychotropic drugs were used by 89% of HIV-infected patients and that anxiolytics and hypnotics were consumed by 49% and 43% of HIV-infected patients, respectively [15].

We inferred from the results that psychiatric illnesses were substantial predictors for BZD and Z-drug users. For any BZD and Z-drug users, insomnia had the highest OR (20.09), whereas, for the long-term users, mood disorder was the leading factor (OR: 15.69).

The association between psychiatric illness and HIV infection has been discussed in previous studies [1, 16]. Mental illnesses in HIV-infected patients can either occur prior to infection, potentially increasing infection risks and strongly predicting future complications such as depression [17], or develop after infection-associated stress, maladjustment, depression, or other psychological problems are encountered [18]. Psychiatric comorbidities among HIV-infected patients have ranged from 20% to 50% in previous studies and have been widely discussed because HIV is considered to be a stigmatized disease that creates considerable psychological and physical stress [1, 19]. In a previous study based on the claims data of a nationally representative cohort from the Taiwan NHIRD during 1998 and 2006, 274 (23.8%) of 1,153 newly diagnosed HIV-infected patients were identified as having psychiatric disorders, the majority of which were anxiety and depressive disorders [20]. Furthermore, psychiatric disorders are associated with risky sexual behaviors and are significant risk factors for HIV infection [18]. Nduna et al. [21] recruited 1,002 female and 976 male volunteers between 15 and 26 years of age in South Africa to examine the influence that psychiatric symptoms and psychosocial stress exerted on their HIV risk behaviors. Their results indicated that women with depression symptoms were highly likely to report transactional sex and intimate partner violence, and men with depression symptoms were associated with a high frequency of transactional sex and failure to use a condom. Nduna et al. [21] suggested that mental health and psychosocial problems including depression, substance use, and violence were associated with increased HIV transmission risk behaviors among the population of men who have sex with men [22].

According to our study result, HIV infection itself is a significant predictor for BZD and Z-drug use. The intense requirement for BZDs and Z-drugs among HIV-infected patients may indicate the clinical complexity of HIV infection. Insomniac symptoms may have resulted from various factors, including physical symptoms of HIV-related diseases, such as opportunistic infections, drug-related insomniac symptoms, adjustment problems, and other clinical concerns.

The results of this study showed that 76.4% of the HIV-infected patients had BZD or Z-drug prescriptions and that psychiatric illnesses were substantial risk factors. However, these prescriptions were primarily prescribed by nonpsychiatrists, and only 31.1% of the patients sought mental health support and received prescriptions from psychiatrists. Although low rates of prescriptions from psychiatrists among the controls (34.3%) were observed, further intensive psychiatric support for HIV-infected patients is required, particularly because previous reports have indicated that BZD use is associated with a low quality of life and high-HIV-risk behaviors among patients with substance use disorders [23]. Therefore, determining the pattern of BZD and Z-drug use among HIV-infected patients and the associated psychiatric factors can facilitate additional detailed clinical evaluations and management.

### 4.1. Study Limitations

Several limitations of this study should be mentioned. The prevalence of psychiatric disorders may be substantially underestimated because the results were derived from insurance data based on ICD diagnostic codes. In addition, most people with HIV receive long-term treatment at clinics from infectious disease physicians who are unfamiliar with psychiatric diagnoses and codes; thus, psychiatric comorbidities are potentially underestimated and overestimated in HIV-infected patients. In addition, the diagnostic accuracy of the psychiatric comorbidities must be considered because this is a database study.

### 4.2. Conclusion

In conclusion, BZDs and Z-drugs are commonly used by HIV-infected patients, and mood disorders, anxiety disorders, substance disorders, insomnia, and HIV infection were significant predictors of drug use. Therefore, additional psychiatric services and mental health support are highly recommended for enhancing the treatment outcome and quality of life for HIV-infected patients.

## Figures and Tables

**Table 1:** Demographic data and prevalence of BZDs and Z-drugs use among patients with HIV and the control group.

	Patients with HIV (*n* = 1081)	Controls (*n* = 1081)	*P* value
Age at HIV infection/enrollment (years, SD)	33.41 (13.81)	33.41 (13.81)	
Sex (male, %)	787 (72.8)	787 (72.8)	
Duration of infection (years, SD)	6.63 (4.09)	—	
Any use of BZDs and Z-drug (*n*, %)	826 (76.4)	417 (38.6)	<0.001
Nonuser	255 (23.6)	664 (61.4)	
BZDs alone user	415 (38.4)	227 (21.0)	
Z-drugs alone user	21 (1.9)	25 (2.3)	
Combination user	390 (36.1)	165 (15.3)	
cDDD of BZDs and Z-drug (SD, *n*, %)	376.15 (1137.35)	140.25 (955.57)	<0.001
<180	751 (69.5)	921 (85.2)	
≧180	330 (30.5)	160 (14.8)	
Psychiatric comorbidities (*n*, %)			
Any mood disorder	140 (13.0)	121 (11.2)	0.235
Any anxiety disorder	155 (13.4)	121 (11.2)	0.033
Alcohol use disorder	9 (0.8)	8 (0.7)	>0.999
Substance use disorder	110 (10.2)	107 (9.9)	0.886
Insomnia	191 (17.7)	167 (15.4)	0.183
Level of urbanization (*n*, %)			0.963
1 (most urbanized)	385 (35.6)	399 (36.9)	
2	324 (30.0)	321 (29.7)	
3	185 (17.1)	174 (16.1)	
4	120 (11.1)	120 (11.1)	
5 (most rural)	67 (6.2)	67 (6.2)	
Income-related insured amount			0.010
≤15,840 NTD/month	457 (42.3)	388 (35.9)	
15,841~25,000 NTD/month	332 (30.7)	370 (34.2)	
≥25,001 NTD/month	292 (27.0)	323 (29.9)	

SD: standard deviation; NTD: New Taiwan Dollar; cDDD: cumulative defined daily dose.

**Table 2:** Predictors of use of BZDs and Z-drugs among HIV-infected patients and the controls.

Characteristics	Any use of BZDs and Z-drugs^*^		Long-term use of BZDs and Z-drugs^∗,#^	
	OR (95% CI)	*P* value	OR (95% CI)	*P* value
HIV, presence versus absence	**8.70 (6.82~10.97)**	**<0.001**	**5.06 (3.63~7.04)**	**<0.001**
Psychiatric comorbidities, presence versus absence				
Any mood disorder	**9.86 (5.37~18.12)**	**<0.001**	**15.69 (10.18~24.17)**	**<0.001**
Any anxiety disorder	**7.24 (3.57~14.67)**	**<0.001**	**6.53 (4.34~9.82)**	**<0.001**
Alcohol use disorder	3.21 (0.72~14.43)	0.128	1.55 (0.29~8.38)	0.621
Substance use disorder	1.26 (0.83~1.92)	0.281	**2.33 (1.49~3.64)**	**<0.001**
Insomnia	**20.09 (10.49~38.47)**	**<0.001**	**10.69 (7.46~15.31)**	**<0.001**

^*^Adjusted for demographic data and psychiatric comorbidities and HIV as a binary variable.

^#^Long-term use of BZDs and Z-drugs means cumulative defined daily dose ≧180.

OR: odds ratio; CI: confidence interval.

**Bold type** indicates the statistical significance.

## References

[1] Bing E. G., Burnam M. A., Longshore D. (2001). Psychiatric disorders and drug use among human immunodeficiency virus-infected adults in the United States. *Archives of General Psychiatry*.

[2] Dubé B., Benton T., Cruess D. G., Evans D. L. (2005). Neuropsychiatric manifestations of HIV infection and AIDS. *Journal of Psychiatry & Neuroscience*.

[3] Israelski D. M., Prentiss D. E., Lubega S. (2007). Psychiatric co-morbidity in vulnerable populations receiving primary care for HIV/AIDS. *AIDS Care—Psychological and Socio-Medical Aspects of AIDS/HIV*.

[4] Martinez J., Hosek S. G., Carleton R. A. (2009). Screening and assessing violence and mental health disorders in a cohort of inner city HIV-positive youth between 1998–2006. *AIDS Patient Care and STDs*.

[5] Twu S.-J., Huang Y.-F., Lai A.-C., Su I.-J. (2004). Update and projection on HIV/AIDS in Taiwan. *AIDS Education and Prevention*.

[6] Rubinstein M. L., Selwyn P. A. (1998). High prevalence of insomnia in an outpatient population with HIV infection. *Journal of Acquired Immune Deficiency Syndromes and Human Retrovirology*.

[7] Hudson A., Kirksey K., Holzemer W. (2004). The influence of symptoms on quality of life among HIV-infected women. *Western Journal of Nursing Research*.

[8] Hudson A. L., Portillo C. J., Lee K. A. (2008). Sleep disturbances in women with HIV or AIDS: efficacy of a tailored sleep promotion intervention. *Nursing Research*.

[9] Vitiello B., Burnam M. A., Bing E. G., Beckman R., Shapiro M. F. (2003). Use of psychotropic medications among HIV-infected patients in the United States. *The American Journal of Psychiatry*.

[10] Lader M. (2014). Benzodiazepine harm: how can it be reduced?. *British Journal of Clinical Pharmacology*.

[11] Darke S., Hall W., Ross M., Wodak A. (1992). Benzodiazepine use and HIV risk-taking behaviour among injecting drug users. *Drug and Alcohol Dependence*.

[12] Victorri-Vigneau C., Gérardin M., Rousselet M., Guerlais M., Grall-Bronnec M., Jolliet P. (2014). An update on zolpidem abuse and dependence. *Journal of Addictive Diseases*.

[13] http://w3.nhri.org.tw/nhird/date_01.html.

[14] Takaesu Y., Komada Y., Asaoka S., Kagimura T., Inoue Y. (2014). Factors associated with long-term use of hypnotics among patients with chronic insomnia. *PLoS ONE*.

[15] Ochitill H., Dilley J., Kohlwes J. (1991). Psychotropic drug prescribing for hospitalized patients with acquired immunodeficiency syndrome. *The American Journal of Medicine*.

[16] Hinkin C. H., Castellon S. A., Atkinson J. H., Goodkin K. (2001). Neuropsychiatric aspects of HIV infection among older adults. *Journal of Clinical Epidemiology*.

[17] Atkinson J. H., Heaton R. K., Patterson T. L. (2008). Two-year prospective study of major depressive disorder in HIV-infected men. *Journal of Affective Disorders*.

[18] Benton T. D. (2008). Depression and HIV/AIDS. *Current Psychiatry Reports*.

[19] Peng E. Y. C., Lee M. B., Morisky D. E. (2010). Psychiatric Morbidity in HIV-infected male prisoners. *Journal of the Formosan Medical Association*.

[20] Chen M.-H., Su T.-P., Chen T.-J., Cheng J.-Y., Wei H.-T., Bai Y.-M. (2012). Identification of psychiatric disorders among human immunodeficiency virus-infected individuals in Taiwan, a nine-year nationwide population-based study. *AIDS Care*.

[21] Nduna M., Jewkes R. K., Dunkle K. L., Shai N. P. J., Colman I. (2010). Associations between depressive symptoms, sexual behaviour and relationship characteristics: a prospective cohort study of young women and men in the Eastern Cape, South Africa. *Journal of the International AIDS Society*.

[22] Safren S. A., Traeger L., Skeer M. R. (2010). Testing a social-cognitive model of HIV transmission risk behaviors in HIV-infected MSM with and without depression. *Health Psychology*.

[23] Chander G., Himelhoch S., Moore R. D. (2006). Substance abuse and psychiatric disorders in HIV-positive patients: epidemiology and impact on antiretroviral therapy. *Drugs*.

